# Dynamic Thermal Treatments in Green Coconut Water Induce Dynamic Stress Adaptation of *Listeria innocua* That Increases Its Thermal Resistance

**DOI:** 10.3390/foods12214015

**Published:** 2023-11-03

**Authors:** Gerardo A. González-Tejedor, Alberto Garre, Asunción Iguaz, Ricardo Wong-Zhang, Pablo S. Fernández, Arícia Possas

**Affiliations:** 1 Sistema Nacional de Investigación (SNI), Senacyt, Ciudad de Panamá, Panama; gerardo.gonzalez@utp.ac.pa; 2 Centro de Producción e Investigaciones Agroindustriales, Universidad Tecnológica de Panamá, Ciudad de Panamá, Panama; ricardo.wong2@utp.ac.pa; 3 Departamento de Ingeniería Agronómica, Instituto de Biotecnología Vegetal, Universidad Politécnica de Cartagena, 30202 Cartagena, Spain; alberto.garre@upct.es (A.G.); asun.iguaz@upct.es (A.I.); 4 Department of Food Science and Technology, UIC Zoonosis y Enfermedades Emergentes ENZOEM, CeiA3, Universidad de Córdoba, 14014 Córdoba, Spain

**Keywords:** inactivation, plant-based beverages, stress response, foodborne pathogens, modelling

## Abstract

The global coconut water market is projected to grow in the upcoming years, attributed to its numerous health benefits. However, due to its susceptibility to microbial contamination and the limitations of non-thermal decontamination methods, thermal treatments remain the primary approach to ensure the shelf-life stability and the microbiological safety of the product. In this study, the thermal inactivation of *Listeria innocua*, a *Listeria monocytogenes* surrogate, was evaluated in coconut water and in tryptone soy broth (TSB) under both isothermal (50–60 °C) and dynamic conditions (from 30 to 60 °C, with temperature increases of 0.5, 1 and 5 °C/min). Mathematical models were used to analyse the inactivation data. The Geeraerd model effectively described the thermal inactivation of *L. innocua* in both TSB and coconut water under isothermal conditions, with close agreement between experimental data and model fits. Parameter estimates and analysis revealed that acidified TSB is a suitable surrogate medium for studying the thermal inactivation of *L. innocua* in coconut water, despite minor differences observed in the shoulder length of inactivation curves, likely attributed to the media composition. The models fitted to the data obtained at isothermal conditions fail to predict *L. innocua* responses under dynamic conditions. This is attributed to the stress acclimation phenomenon that takes place under dynamic conditions, where bacterial cells adapt to initial sub-lethal treatment stages, leading to increased thermal resistance. Fitting the Bigelow model directly to dynamic data with fixed *z*-values reveals a three-fold increase in *D*-values with lower heating rates, supporting the role of stress acclimation. The findings of this study aid in designing pasteurization treatments targeting *L. innocua* in coconut water and enable the establishment of safe, mild heat treatments for refrigerated, high-quality coconut water.

## 1. Introduction

Coconut water is the sweet and transparent fluid that can be found inside young coconuts (*Cocos nucifera* L.) [1]. This plant-based product is mainly composed of water (around 95%), and also contains sugars such as sucrose, glucose or fructose and small amounts of proteins and lipids [2,3]. It also contains calcium, magnesium, potassium, sodium or phosphorus, among other inorganic ions, as well as vitamins such as vitamin C, thiamine, riboflavin, niacin, pantothenic acid, pyridoxine and folates [4].

The global coconut water market is expected to grow with a compound annual growth rate of 10.8% from 2023 to 2028 [5]. As a plant-based product, it has gained popularity in recent years, offering a myriad of health benefits to consumers. The nutrients present in coconut water include active components that could prevent oxidative stress [6,7]. Moreover, this refreshing drink possesses anti-inflammatory activity, cardio-protectant properties, and has shown to improve the lipidic profile and reduce high blood pressure [8,9,10,11]. It has also been used for oral rehydration, besides being proposed as an intravenous solution to hydrate patients in emergency situations [10,12].

Due to its nutritious and physicochemical characteristics (e.g., pH 5.6), once the water is extracted from coconuts, it can be easily deteriorated by the activity of microorganisms and enzymes [13]. Additionally, this beverage can become contaminated with bacterial pathogens, such as spore-forming bacteria, *Salmonella* spp. and *Listeria monocytogenes*, during harvest, since coconuts are usually placed on the soil, and during handling and extraction [14]. Once contaminated, these pathogens could persist over the coconut shelf-life. For instance, *L. monocytogenes* has shown great growth capacity in the product at temperatures ranging from 4 to 35 °C [14].

Different non-thermal decontamination techniques for coconut water have been investigated, including pulsed light [15], UV-C light [16], atmospheric cold plasma [17] and high hydrostatic pressure, among others [13]. These non-thermal treatments have shown different levels of effectiveness, but experience limitations for their commercial implementation, such as high costs and scaling-up [13]. Despite the negative effects of thermal treatments on coconut water quality, they remain as the most applied method to mitigate microbial contamination in this beverage, mainly for yielding shelf-stable products [2,15].

In most industrial heat treatment processes, foods are subjected to dynamic heating, meaning that temperatures are not immediately raised from room to pasteurization values [18]. Mathematical models have been applied to describe *L. monocytogenes* thermal inactivation kinetics in different food products under isothermal and dynamic conditions, reflecting real life scenarios. In addition, the empirical research has demonstrated that models relying on isothermal data frequently fail in accurately predicting microbial inactivation when dynamic conditions are involved [19,20].

In situations involving dynamic heating, microbial populations may adapt to stress conditions [18,21]. This adaptation involves bacterial cells activating a range of stress response mechanisms that enhance their chances of surviving. When the heating rate is slow, these mechanisms may kick in during the early stages of dynamic treatment, leading to an adaptation to stress and greater resistance to the lethal phase of treatment than what would be anticipated based solely on isothermal experiments [18].

Therefore, this study develops a dynamic model to predict the response of *L. monocytogenes* during the pasteurization of coconut milk, designed to be combined with subsequent refrigeration. The model is based on *Listeria innocua*, a common surrogate for *L. monocytogenes* [22,23]. Due to its ability to predict the number of bacterial reductions for different temperature profiles, accounting for dynamic stress acclimation, the model can be a valuable tool for the design of thermal pasteurization treatments for this product.

## 2. Materials and Methods

### 2.1. Substrate

Coconuts (*Cocos nucifera*) were obtained from a local producer from Panama. They were washed with chlorinated water, and coconut water was extracted mechanically with a tool that perforated the external parts and allowed for the extraction. It was collected in a sterile recipient and used directly for heat resistance experiments or stored in a refrigerator until use.

### 2.2. Microorganism and Preparation

*Listeria innocua* serovar 6a (CECT 910) was obtained as a lyophilized culture from the Spanish Type Culture Collection following the methodology previously described [24]. A stock containing approximately 10^9^ CFU/mL was prepared. It was mixed with glycerol (30%) and kept frozen at −80 °C until use.

To perform the experiments, *L. innocua* was grown overnight from the frozen stock, adding 200 μL to 50 mL of Tryptone Soya Broth (TSB; Scharlab Chemie S.A., Barcelona, Spain). It was then incubated for 12 h to reach the stationary phase with a concentration of 10^8^ CFU/mL, that was used as inoculum for heat resistance experiments. The cell concentration was confirmed by viable plate counts, performing decimal dilutions in peptone water, and growing the samples in Tryptone Soya Agar (TSA; Scharlab Chemie S.A., Barcelona, Spain) for 48 h at 37 °C. Plates containing between 30 and 300 CFU were then counted.

### 2.3. Microbial Inactivation Experiments

A 400 mL volume of TSB or coconut water was introduced in the vessel of a thermoresistometer Mastia [25], previously sterilized, and heated at the target temperature. Then, a 200 μL volume of the *L. innocua* suspension was injected with a Hamilton pipette into the vessel and samples were taken at the time intervals selected. The temperatures for isothermal inactivation experiments were 50, 52.5, 55, 57.5 and 60 °C. For non-isothermal conditions, ramps starting at 30 °C up to 57.5 or 60 °C were programmed, with temperature increases of 0.5, 1 and 5 °C/min. Samples were cooled immediately and viable plate counts were performed as explained above.

### 2.4. Microbial Inactivation under Isothermal Conditions

The data on microbial inactivation of *L. innocua* on both media were analysed using the Geeraerd model without a tail [26] due to the nonlinearity of the survivor curves. This model describes the relationship between the microbial concentration (*N*) and the treatment time (*t*), as shown in Equation (1), where *SL* is the shoulder length and *k* is the inactivation rate during the exponential phase.

(1) $$N=N_{0}\cdot e^{-k\cdot t}\cdot e^{k\cdot SL}/(1+(e^{k\cdot SL}-1)\cdot e^{-k\cdot t})$$

To ease the interpretation, the models were fitted using the *D*-value (*D*), which represents the treatment time required to reduce the microbial concentration by 1 decimal logarithm, instead of the inactivation rate (*k*), using the identity *D* = ln(10)/*k*.

We used a Bigelow-type secondary model to describe the relationship between the *D*-value and the treatment temperature, as shown in Equation (2). This model introduces a reference temperature (*T_ref_*) that improves the parameter identifiability but without any biological meaning. This parameter was set at the mean of the temperature range (55 °C), as previously suggested [27]. Then, this model is defined by the *D*-value at the reference temperature (*D_ref_*) and the *z*-value (*z*), which defines the temperature increase required to reduce the *D*-value by 90%.

(2) $$logD=logD_{ref}-\frac{T-T_{ref}}{z}$$

The relationship between the shoulder length duration (*SL*) and the storage temperature was described using the model from Equation (3), where *a* and *b* are two unknown regression coefficients.

(3) $$logSL=a-b(T-T_{ref})$$

Primary inactivation models were fitted to the isothermal data using the functions included in the *bioinactivation* package for R [28], using its web interface [29] currently available at: https://foodlab-upct.shinyapps.io/bioinactivation4/ (accessed on 4 August 2023). Then, the relationship between the model parameters and the treatment temperature was evaluated visually to suggest secondary models. Finally, a global inactivation model was fitted using a one-step fitting algorithm to the complete isothermal dataset, as this approach often provides more accurate parameter estimates than the two-step method [30]. The global model was fitted using R version 4.2.3 [31] using the Levenberg–Marquard algorithm [32] implemented in the *minpack.lm* package version 1.2-3 [33].

### 2.5. Microbial Inactivation under Dynamic Heating Conditions

Microbial inactivation under dynamic conditions was studied using both the Bigelow and Geeraerd inactivation models. The Bigelow model (Equation (4)) is a generalization of the Bigelow model for isothermal conditions (Equation (1)), so it assumes first-order inactivation kinetics. The inactivation rate is quantified by the *D*-value (*D*), whose relationship with temperature is given by the same secondary model as for isothermal conditions (Equation (2)).

d*N*/d*t* = −ln(10)/*D*(*T*)·*N* (4)

The Geeraerd inactivation model [24] is an extension of first-order kinetics based on similar arguments as the Baranyi growth model [34]. As shown in Equation (5), it introduces two correction factors in the differential equation: one to describe the shoulder (*α*) and one for the tail (*β*).

d*N*/d*t* = −*α·k*·*β·N* (5)

The shoulder is supported on a theoretical substance (*C*) that must be inactivated before bacterial inactivation takes place. In this model, it is assumed that *C* follows first-order kinetics (Equation (6)) and affects the population inactivation rate as shown in Equation (7). Accordingly, the shoulder length is defined by the initial value of this theoretical substance (*C*_0_) by the identity shown in Equation (8).

d*C*/d*t* = −*k*·*C* (6)

*α* = 1/(1 + *C*) (7)

*SL* = (ln (*C*_0_) + 1)/*k* (8)

The tail is described in the Geeraerd model assuming that there is a horizontal asymptote on the bacterial concentration (*N_res_*) and that the population is self-regulated by a logistic term (Equation (9)).

*β* = (1 − *N_res_*/*N*) (9)

The parameter values estimated from isothermal conditions (Section 2.4) were used for making predictions under dynamic conditions, comparing them against the experimental data. This was carried out using the web version of *bioinactivation* (available at https://foodlab-upct.shinyapps.io/bioinactivation4/, accessed on 4 August 2023), which solves the differential equation numerically. Due to the lack of a tail in the experimental data, parameter *β* was fixed to one. Regarding the shoulder length, it is not clear how isothermal results can be extrapolated to dynamic conditions, due to the latter being defined by *C*_0_ (and different *SL* resulting in different *C*_0_; Equation (8)). Consequently, predictions were calculated for the maximum and minimum values of *C*_0_ corresponding to the shoulder lengths estimated from the isothermal data.

Moreover, to further analyse the differences between predictions based on isothermal conditions and dynamic observations, the dynamic Bigelow model was fitted to the dynamic observations using the functions included in *bioinactivation.* To simplify comparison against isothermal models, the models were fitted fixing the *z*-value to the one estimated from isothermal conditions.

## 3. Results and Discussion

### 3.1. Inactivation of Listeria innocua in TSB and Coconut Water under Isothermal Conditions

**Figure 1:**
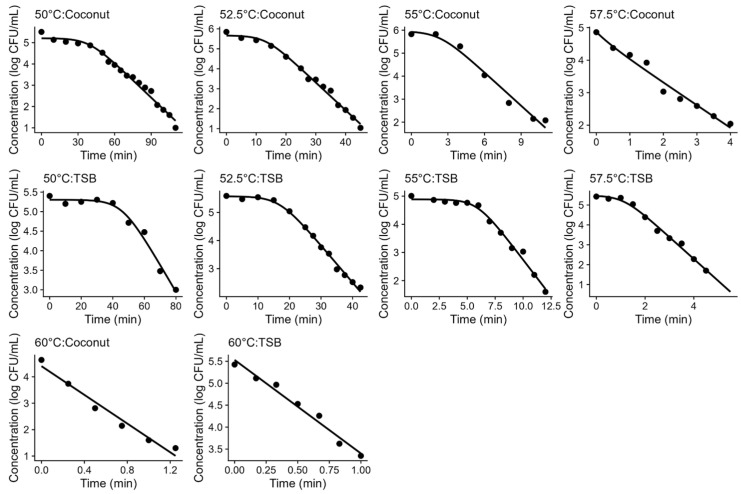
Fitting of the Geeraerd model (solid line) to the inactivation data of *Listeria innocua* (•) under isothermal conditions. The subplots show the results at different temperatures for acidified TSB and coconut water (conditions showed in titles).

The Geeraerd model was successful at describing the thermal inactivation of *L. innocua* in both TSB and coconut water under isothermal conditions at all the temperatures tested. Figure 1 illustrates both the experimental data and the fitted models, showing a good agreement between the data and model fits. These plots also show that the inactivation kinetics in TSB were similar to those in coconut water, evidencing that acidified TSB could be a good surrogate medium for the thermal inactivation of *L. innocua* in coconut water.

**Figure 2:**
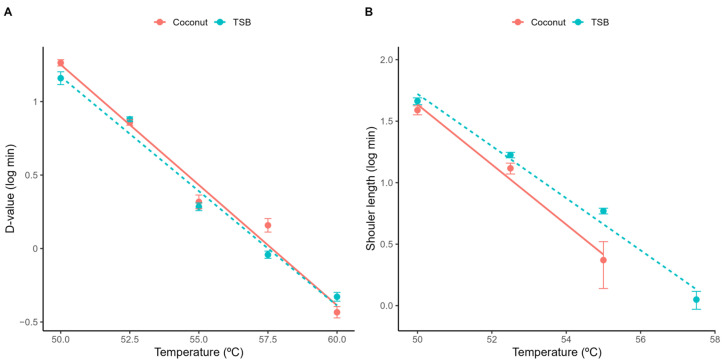
Parameter estimates of the Geeraerd model fitted to each individual isothermal inactivation experiment. *D*-value (**A**) and shoulder length (**B**).

The similarities between the media are further confirmed in Table 1, which provides the parameter estimates of the primary models fitted to each isothermal condition. These parameters are illustrated in Figure 2, showing that there is practically no difference between the *D*-values obtained in both media (Figure 2). Although there is a small difference in the shoulder length observed in coconut water and acidified TSB (with the laboratory media showing slightly longer shoulder lengths than the food product), these results also support TSB as a good surrogate for thermal inactivation of *L. innocua* in coconut water, as models based on TSB would provide conservative predictions (“fail-safe”).

These differences in shoulder length are likely due to the composition of the media. The shoulder is often interpreted as the representation of thermal inactivation being a “multi-hit” process, with the microbial cells being able to resist the treatment for some time before inactivation is effective [35]. This resistance is often linked to the physiological state of cells (e.g., membrane porosity), which is dependent on the media composition [36,37]. Therefore, the composition of acidified TSB (which is richer than coconut water) is likely to contain elements that increase the thermal resistance of *L. innocua*.

Figure 2 also illustrates the relationship between the model parameters of the Geeraerd model and the treatment temperature. As expected, the *D*-value showed a log-linear relationship with temperature, as is common in this type of model (Figure 2A). Moreover, the shoulder length also showed a log-linear trend with temperature (Figure 2B). This result is interesting, because the shoulder length is often considered more complex than the *D*-value, as it often depends on additional factors such as the cell history.

The log-linear relationships illustrated in Figure 2 justify the development of log-linear secondary models for both parameters. Table 1 provides the parameter estimates of these secondary models fitted to the complete dataset (for each media) using a one-step model fitting approach. The model parameters agree with the previous interpretation regarding media effects. The estimates of *z* and *D_ref_* are practically the same on both media, confirming that the inactivation rate during the exponential phase in acidified TSB is practically the same as for coconut water. On the other hand, the intercept of the secondary model for the shoulder length (*a*) is higher in acidified TSB than in coconut water, whereas the slope (*b*) is lower, indicating that the shoulder length is longer on acidified TSB than in coconut water. Therefore, acidified TSB would be an adequate surrogate media to study the isothermal inactivation of *L. innocua* in coconut water.

### 3.2. Inactivation of Listeria innocua in TSB and Coconut Water under Dynamic Conditions

**Figure 3:**
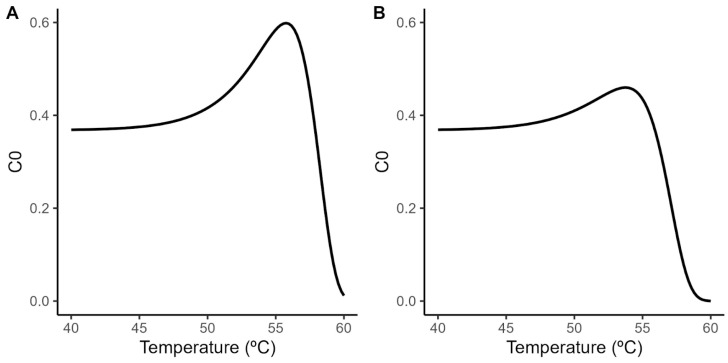
Relationship between initial value of the theoretical substance *C* and the treatment temperature according to the secondary model for the shoulder length (Equation (3)) and the relationship between both variables (Equation (7)) in acidified TSB (**A**) and coconut water (**B**).

A principal challenge when using the Geeraerd model is the extrapolation of the shoulder length estimated under isothermal conditions to dynamic heating conditions. Although the relationship between the shoulder length and the treatment temperature has been successfully described using a log-linear model (Figure 2; Equation (3)), dynamic predictions require the definition of the initial value for the ideal substance *C* (*C*_0_). Despite the relationship between *SL* and C_0_ being well-defined by the identity in Equation (7), combining this equation with the secondary models for *SL* and *D* (Equations (2) and (3)) results in the nonlinear relationship between C_0_ and the treatment temperature illustrated in Figure 3. This raises the question of what value of *C*_0_ to use for the model predictions, as the Geeraerd model requires the definition of a unique value for this parameter.

In this study, we use the maximum (*C*_0_ = 0.6 in acidified TSB; *C*_0_ = 0.46 in coconut water) and minimum estimated values (*C*_0_ = 0; equivalent to the Bigelow model) as a way to generate a reasonable envelope for the microbial response based on the experimental data. These values are combined with the parameters of the secondary model for the temperature (Table 1) to predict the microbial response under dynamic environmental conditions.

**Figure 4:**
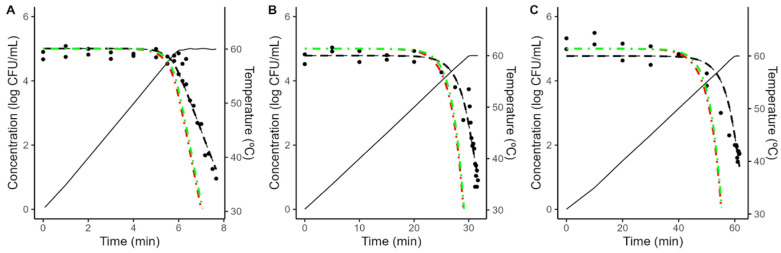
Comparison between the predictions of the Geeraerd model fitted to isothermal data considering the maximum possible value of *C*_0_ (green, dash-dot line) no shoulder (red, dash-dot line) against the experimental data (•) obtained during dynamic heating (black, solid line) in acidified TSB with a heating rate of 5 (**A**), 1 (**B**) or 0.5 (**C**) °C/min. The plots also show the fit of the Bigelow model fitted directly to the dynamic data (black, dashed line).

**Figure 5:**
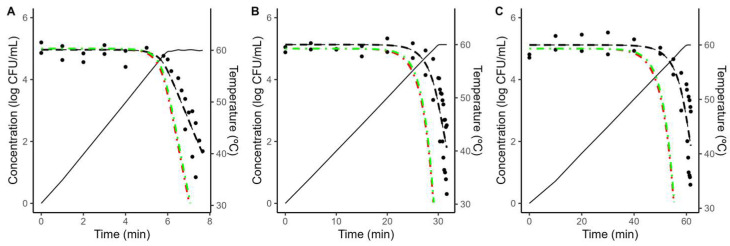
Comparison between the predictions of the Geeraerd model fitted to isothermal data considering the maximum possible value of *C*_0_ (green, dash-dot line) no shoulder (red, dash-dot line) against the experimental data (•) obtained during dynamic heating (black, solid line) in coconut water with a heating rate of 5 (**A**), 1 (**B**) or 0.5 (**C**) °C/min. The plots also show the fit of the Bigelow model fitted directly to the dynamic data (black, dashed line).

As illustrated in Figure 4 and Figure 5, the models fitted to isothermal conditions fail to predict the microbial response under dynamic heating. This deviation between predictions based on isothermal experiments and results under dynamic conditions is often reported in the scientific literature and can be attributed to various mechanisms [20,38,39,40,41].

One possibility is arguing for an initial bacterial resistance, using similar arguments as for the shoulder in isothermal inactivation curves. However, as illustrated in Figure 4 and Figure 5, even when the shoulder is introduced using the worst-case scenario for *C*_0_, the effect on the model predictions is minimal. Hence, an initial bacterial resistance is unlikely to be the cause for the deviation between predictions based on isothermal data and dynamic observations.

An alternative justification that has recently gained interest within the scientific community is the phenomenon known as “stress acclimation”. This hypothesis considered that bacterial cells adapt dynamically to the initial (sub-lethal) stages of the treatment, activating a stress response that increases their thermal resistance [18,42,43]. Stress acclimation is often evidenced by lower heating rates resulting in a large deviation between the model predictions and the observations, due to the bacterial population having more time to adapt to the treatment.

To obtain further insight into the microbial response under dynamic conditions, the Bigelow model was fitted directly to the dynamic data, fixing the *z*-value to the one obtained under dynamic conditions to simplify the comparison between different conditions. As illustrated in Figure 4 and Figure 5, the model fitted to dynamic conditions is able to describe the overall trend in the microbial population. Table 2 reports the parameter values estimated for each condition. It shows that a reduction on the heating rate from 5 to 0.5 °C/min results in approximately a three-fold increase in the *D*-value (5.66 to 17.99 min in acidified TSB; 6.23 to 18.77 min in coconut water).

This result is compatible with the assumption that stress acclimation would be responsible for the differences in the response of *L. innocua* under isothermal and dynamic conditions, as lower heating rates would allow higher stress acclimation due to cells being exposed to sublethal temperatures for a longer time.

The parameters reported in Table 2 provide further arguments to consider acidified TSB as a suitable surrogate for the thermal inactivation of *L. innocua* in coconut water. Even in dynamic treatments where stress acclimation seems relevant, the *D*-values obtained in acidified TSB are practically the same as those observed in coconut water. This, combined with the results observed under isothermal conditions, confirms that this media can be used as a reliable method for the design of mild pasteurization treatments targeting *L. innocua* in this product. They would need to be combined with refrigeration to inhibit the growth of high heat-resistant, spore forming bacteria.

The comparison between the *D*-values obtained under isothermal and dynamic conditions showed comparable results to those obtained in similar studies. Considering that isothermal experiments resulted in an estimate of the *D*-value of 2.88 min at 55 °C (Table 1), the lowest heating rate would induce a ~6.5 fold increase in the *D*-value. This value is comparable to those reported by Clemente-Carazo et al. [44], who reported a ~7 fold increase in the *D*-value of three strains of *L. monocytogenes* under dynamic conditions on buffered TSB and milk. This result is of high relevance due to the use of a different bacterial strain and media in this study (in fact, a media of different pH), as it could indicate that there could be an overall maximum stress acclimation attainable by a bacterial population that would be independent of the bacterial strain and media. Nonetheless, this hypothesis would need to be studied further in the future.

While non-thermal methods have proven effective in reducing bacterial pathogens in coconut water [13,15,16,17], it is worth noting that these investigations were conducted on a small laboratory scale (volumes of 5–10 mL) and employed varying process parameters. Therefore, a direct comparison of their efficacy with the current study, which simulates industrial dynamic treatment conditions, is not feasible.

Although the model is based on experimental data for *L. innocua*, there is scientific evidence of this microbial species being a suitable surrogate for the thermal inactivation of *L. monocytogenes* [22]. Hence, the model will be a useful tool to establish safe, mild heat treatments for coconut water. It could also be the basis for future studies that determine optimum heating profiles using a multifactorial approach that combines food safety with nutritional and environmental aspects.

## 4. Conclusions

Mild heat treatments can be an alternative to preserve high quality coconut water, but a careful design of the heating conditions, in order to avoid microbial stress adaptation, should be carefully selected. Precise knowledge of heat inactivation parameters modelled appropriately is a key aspect for designing safe thermal treatments. This research underscores the need for specific modelling approaches to account for stress acclimation under dynamic temperature conditions when targeting the inactivation of *Listeria* spp., facilitating the design of safe pasteurization processes for coconut water.

## Figures and Tables

**Table 1:** Parameter estimates (estimate ± standard error) for the Geeraerd model with log-linear secondary models for *D* and *SL* fitted to the isothermal inactivation of *Listeria innocua* in acidified TSB and coconut water using a one-step approach.

Parameter	Acidified TSB	Coconut Water
log *N*_0_ (log CFU/mL)	5.34 ± 0.11	5.30 ± 0.23
*a* (min)	0.58 ± 0.07	0.12 ± 0.31
*b* (min/°C)	0.20 ± 0.01	0.23 ± 0.05
log *D*_55_ (log min)	0.46 ± 0.03	0.46 ± 0.04
*z* (°C)	4.94 ± 0.25	5.00 ± 0.24

**Table 2:** Estimates of the *D*-value at 55 °C (estimate ± standard error) from data obtained under dynamic heating conditions with different heating rates. To ease the interpretation, the *z*-value was fixed to the one obtained under isothermal conditions (4.99 °C on acidified TSB, 5.00 °C in coconut water).

Medium	Heating Rate (°C/Min)	*D*_55_ (Min)
Acidified TSB	0.5	17.99 ± 1.17
	1	10.44 ± 0.07
	5	5.66 ± 0.23
Coconut water	0.5	18.77 ± 2.15
	1	11.64 ± 1.10
	5	6.23 ± 2.04

## Data Availability

The data used to support the findings of this study can be made available by the corresponding authors upon request.
